# Early Microjet Experimentation with Liquid Water in Vacuum

**DOI:** 10.1021/acs.accounts.2c00739

**Published:** 2023-01-31

**Authors:** Manfred Faubel

**Affiliations:** Max-Planck-Institut für Dynamik und Selbstorganisation Bunsenstrasse 10, 37077 Göttingen, Germany

## Abstract

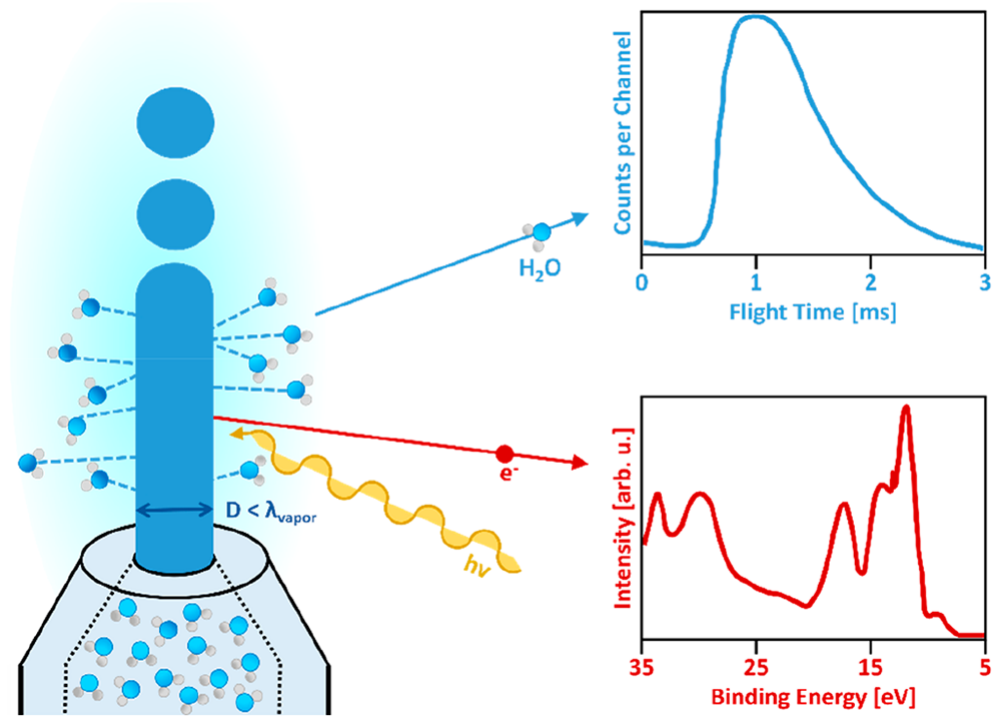

In this brief look at the history of liquid microjets, I recollect some personal reminiscences on initial challenges for introduction of this method, as well as unexpected problems and exemplary results using this new tool for liquid evaporation and photoelectron spectroscopy studies.

Many efficient and direct, atomic level diagnostic instruments in use at solid state surfaces and in gas-phase atom or cluster studies require high vacuum. They have therefore not been applied to investigations of aqueous solutions because liquid water both strongly evaporates and rapidly freezes in vacuum. Only fairly recently, over the past three decades, have liquid microjets been considered as practicable targets for research on liquid-water interfaces in vacuum. The working principle is analogous to the functioning of a free molecular beam source, where molecules enter through a small aperture into a vacuum without being disturbed by subsequent collisions in their original Maxwellian velocity distribution. Similarly, above a microjet surface in vacuum, water vapor molecules do not interact with each other, or with different probe particles, as long as the liquid jet diameter is small in relation to the mean free path of the liquids’ vapor at equilibrium conditions. For pure liquid water, this constraint is *D*_jet_ < λ_vap_ < 10 μm for 6.1 mbar vapor pressure at the triple point of water. A high streaming velocity of the liquid jet, >50 m/s, delays freezing and exposes a steadily renewed fresh vacuum surface for experiments.

For experimental verification of the microjet free surface concept, H_2_O vapor velocities were measured in a molecular beam time-of-flight experiment. These studies showed Maxwellian velocity distributions with the expected local water-jet temperatures for 5 and 10 μm jets, whereas larger liquid jet diameters of 50 μm exhibit narrowed vapor velocity profiles. This narrowing is the known signature of incipient, collision dominated, supersonic hydrodynamic expansions in nozzle beam sources. As a completely unexpected new result in evaporation studies of carboxylic acid solutions, freely evaporating acetic acid dimers showed apparent non-equilibrium liquid surface source temperatures several hundred kelvin above the simultaneously measured monomer temperatures, a phenomenon shown to be correlated with surface tension.

Continuing with improvements, the vacuum water microjets were implemented inside a photoelectron spectroscopy apparatus that was modified for handling large amounts of water vapor. After initial complications with liquid jet charging phenomena, the first partial liquid-water photoelectron spectra were recorded using 21 eV photons from a He I discharge lamp. In the next step, the equipment was taken to a synchrotron radiation beamline at BESSY II, resulting in substantial improvements of signal intensity and in photon tunability for narrow band monochromatic soft X-rays up to 1 keV. Two early examples of these continuing experiments are considered, briefly, for aqueous alkali halide salt solutions and for the pH-value dependent protonation of an NH_2_/NH_3_^+^ group in an amino acid directly in a photoelectron spectrum of a solution.

In conclusion, liquid microjets have opened up a completely new approach to studies of arbitrary liquids with chemical and biological relevance.

## Key References

- SchlemmerS.; FaubelM.; ToenniesJ. P.A Molecular Beam Study of the Evaporation of Water from a Liquid Jet. Z. Physik D: At., Mol. Clusters1988, 10, 269–277.^1^*The existence of a free vacuum surface at micrometer size volatile liquid water jets is demonstrated by vapor molecule velocity measurements, showing for jet diameters D < 10 μm the collisionless propagation of evaporating molecules with a Maxwellian velocity distribution.*
- FaubelM.; KistersT.Non-Equilibrium Molecular Evaporation of Carboxylic Acid Dimers. Nature1989, 339, 527–529.^2^*Non-equilibrium surface temperature increases by >100 K are found in the velocity distribution of dimers emitted from acetic acid–water solutions while CH_3_COOH and water monomers show consistent surface source temperatures, due to surface tension forces acting on evaporating dimer inclusions.*
- WinterB.; WeberR.; HertelI. V.; FaubelM.; JungwirthP.; BrownE. C.; BradforthS. E.Electron Binding Energies of Aqueous Alkali and Halide Ions: EUV Photoelectron Spectroscopy of Liquid Solutions and Combined Ab Initio and Molecular Dynamics Calculations. J. Am. Chem. Soc.2005, 127, 7203–72141588496210.1021/ja042908l.^3^*Vertical and adiabatic ionization energies for solvated anions and cations of all common alkali and halide species are determined by soft X-ray photoelectron spectroscopy.*
- AzizE. F.; OttossonN.; FaubelM.; HertelI. V.; WinterB.Interaction between liquid water and hydroxide revealed by core-hole de-excitation. Nature2008, 455, 89–911876943710.1038/nature07252.^4^*The electronic structures of liquid water and OH^–^_aq_ are obtained by X-ray photoelectron and resonant Auger electron spectroscopy, revealing the inner and valence shells as well as excited electronic states of water and CTTS states of OH^–^_aq_ ions.*

Liquids in vacuum have been investigated and used for a long time in equipment such as vacuum diffusion pumps, in vacuum molecular distillation processes for sensitive chemicals, and in “environmental” electron microscopy. However, studies of aqueous liquid solutions of chemical and biological relevance in vacuum using electron, ion, or molecular beams for high resolution data gathering at the atomic level were not considered seriously as an option for studying chemical processes occurring in the glass test tubes of chemical work benches. Because of its “high vapor pressure”, water was viewed as a liquid that would instantaneously freeze in vacuum into ice. A few early examples of liquid studies in high vacuum were reported for vacuum compatible liquids: in the 1930s for electron diffraction on a mercury droplet and, later, by Siegbahn in the 1970s for X-ray photoelectron spectroscopy on low vapor pressure solvents and on cooled salt solutions.^5,6^

For experiments handling pristine water as an object of observation in high vacuum, a “new” approach, microjets, was not developed until the 1980s. At that time, I had been involved for more than ten years in molecular beam scattering experiments at the Max Planck Institute for Flow Research (MPIfS) in Göttingen, dedicated to investigating fundamental collision processes of atom or ions with simple molecules and with ultraclean crystal surfaces.^7^ Liquids were not an issue, for the stated reasons of handling them in vacuum and, more importantly, perhaps, for lack of an adequate molecular description of liquids. Nevertheless, it was an unspoken challenge of how, and whether at all, aqueous solutions could be made a target for molecular beam type experiments. In designing a new gas phase scattering equipment, I got deeply involved in improving “nozzle beams”, a new technique for generating molecular beams with supersonic, narrow, molecular velocity distribution and with a hundred-fold intensity in comparison with classical, Otto Stern-type, molecular beam oven sources.^8−10^

In supersonic jet expansions of a high-pressure gas, molecules emerge from a small nozzle into vacuum, undergoing many collisions while passing through the nozzle throat and in the initial section of vacuum expansion along radially diverging stream lines. The gas density is then diluted to an extent that further gas molecule collisions become increasingly unlikely, and the dense gas expansion flow changes into a collision free “molecular beam”. In contrast, in traditional “molecular beam sources”, the gas mean free path λ ≈ 1/(σ_collision_·*n*_gas_) is kept larger than the diameter of the gas chambers’ exit aperture into the vacuum. Thus, gas molecules passing through the exit diaphragm will not undergo any subsequent collisions in the vacuum space and form a free molecular beam, in accordance with Stern’s 1919 interpretation of Martin Knudsen’s earlier published theory of gas flows in vacuum.^11^ With this molecular beam functionality in mind, it is immediately obvious that an evaporating liquid water surface will be a free vacuum surface, accessible without any collisions in the emerging vapor gas phase, provided that the lateral extension *D*_λ_ of the liquid surface area is smaller than the equilibrium vapor mean free path λ_H_2_O_. This constraint yields the criterion for a maximum liquid water surface extension of *D*_λ_ ≤ λ_H_2_O_ ≈ 10 μm, the mean free path at a vapor pressure of 6.1 mbar at the freezing point of pure water.^1,2^ The small target area and associated small scattering signals did not appear prohibitively small from the point of view of gas phase scattering experiments.

**Figure 1:**
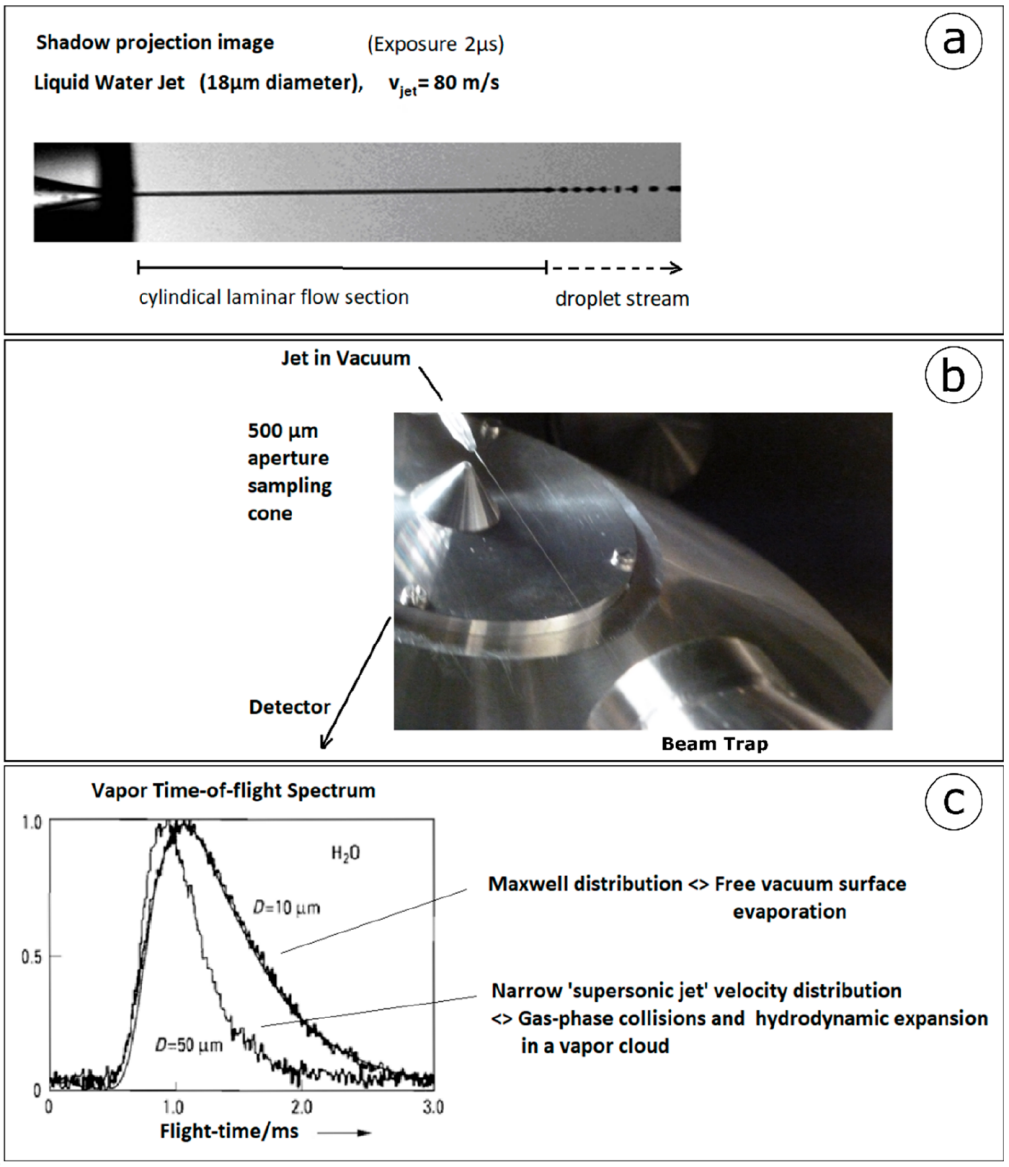
The free surface of liquid water is accessible on a microjet: (a) a high speed, shadow graph picture, at 5 ns exposure, shows an 18 μm diameter water jet flowing out of an 80-bar high pressure nozzle with a velocity of 120 m/s. The laminar flow propagates for several millimeter length as a smooth and continuous cylindrical stream before decaying into regularly spaced droplets. (b) The high-speed liquid microjet passes through a 10^–5^ mbar vacuum chamber. At the detector entrance to ultrahigh vacuum, a conical aperture tip, 0.3 mm wide, samples the vapor effusion (or the photoelectrons) from the smooth, shiny section of the jet between 0.5 and 2 mm distance from the liquid surface. (c) Molecular beam time-of-flight spectra of the emerging vapor from a 5 μm jet and from a 50 μm jet show the distinct differences in the Mach number of the respective velocity distributions. While the narrower velocity distribution of the 50 μm jet shows a Mach number larger than 1, characteristic for molecular beams originating in a hydrodynamic supersonic nozzle expansion, the 5 μm jets’ vapor velocity distribution is Maxwellian, providing a direct proof of the collisionless, interaction free passage of nascent vapor from the 5 μm free liquid water surface into the vacuum. Parts of this figure were reproduced with permission from ref (1). Copyright 1988 Springer.

The exposure of a tiny area of liquid water to high vacuum leads to a number of crucial technical problems, such as how to control the small size of the liquid surface, how to prevent instant freezing of the evaporating liquid, and how to handle the expected gas loads of water vapor on vacuum pumps. Closer estimates show, in free vacuum evaporation, that a water surface at 25 °C ablates at a rate on the order of 0.5 g H_2_O/(cm^2^·s), resulting in evaporative cooling of ∼1 kW/cm^2^. A 10 μm size droplet, thus, is cooled by ∼1 °C in 1–10 μs. In order to run continuous experiments, this requires replacement of the liquid surface target area by a new sample at a flow rate of 10 to 100 m/s, equivalent to shifting the “used” surface by 10 to 100 μm in 1 μs. It turns out that this can be achieved conveniently by operating a liquid jet of water emerging from a small nozzle using a pressure reservoir at ∼1 to 100 bar. In a fortunate coincidence of the laws of physics, the very fast streaming, but very thin, 10 μm liquid jet of water at 100 m/s flows turbulence-free and completely smoothly at laminar flow conditions because it has a Reynolds number far below the critical value for the onset for turbulent flow. Pictured by a nanosecond-flash micrograph in Figure 1a, the surface of an 18 μm diameter microjet is perfectly circular and ripple free when it leaves the nozzle orifice. Only with time and after some distance, the cylindrical liquid filament starts to transform into a stream of droplets, driven by surface tension instabilities, as was first theoretically analyzed by Rayleigh (1879). Rayleigh’s formulas show that the decay depends on liquid properties (density, surface tension, viscosity) and on the liquid filament radius only, but not on flow velocity, as long as the speed is low enough for it not to enter turbulent flow regimes of instability. Thus, the useful length of the cylindrical filament to the point of decay of the liquid string, can be “stretched” outward with higher velocities, up to about 3 mm length for 10 μm jets of lean water, increasing to 3 cm for 20 μm. The initial section of the liquid microjet is a perfectly smooth, stationary cylindrical object with surface roughness limited only by thermal capillary waves to ∼3 Å for pure liquid water.^1,17^

For a typical microjet, the total water flow rate is 0.01 to 0.1 mL/s. Most of it can be removed from the vacuum system by freezing the directed water jet on a liquid nitrogen cold trap or, alternatively, by jet recycling in liquid form, using a catcher assembly with gently heated 50 μm diameter entrance.^12^ The unavoidable loss by jet evaporation of typically 0.5 mbar L/s H_2_O-vapor is pumped away with 5000 L/s cold traps. Figure 1b shows a complete microjet target setup, including a liquid beam catcher, for arbitrary chemical solutions. The conical shaped skimmer aperture samples vapor or scattered electrons from the water jet in vacuum. This initially new and unknown technique for stable handling of liquid microjets in vacuum was tested and improved in a modified molecular beam time-of-flight apparatus. For easier maintenance the liquid jet was placed in a small, separate chamber together with the liquid N_2_ cryotrap.

For velocity distribution analysis, the molecular vapor was sampled at a right angle from the liquid jet with a conventional molecular beam skimmer. In a separate high-vacuum stage, the sampled vapor beam was chopped by a rotating slotted wheel and detected by a fast response mass spectrometer analyzer over an ∼80 cm distance.^8^ Time-of-flight spectra of “nascent” evaporated water, in Figure 1c, show the anticipated Maxwellian velocity distribution for evaporation from 6 to 10 μm wide, “sub-mean-free path” jets, with fitted Maxwell distribution source temperatures in reasonable agreement with evaporating jet cooling model calculations. Gratifyingly, the measured vapor distributions from thicker, 50 μm diameter wide, water jets show significantly narrower, supersonic-jet velocity distributions, providing proof-of-principle evidence for the liquid microjet design concept for preparation of a free-vacuum-surface of water in high and ultrahigh vacuum environments.^1^

Subsequent extension of this study showed that dimers of H_2_O do not directly evaporate from the liquid surface, although they are abundant in supersonic molecular beams.^1,2^ However, the stronger bound dimers of carboxylic acids (*E*_dim_ ∼0.5 eV) show up in large fractions, of >20%, in evaporation from liquid solutions of formic acid, acetic acid, or propionic acid. Very unexpectedly, the dimer nascent velocity distributions are narrower, supersonic-like, functions with apparent source temperatures several 100 K higher than those of the simultaneously measured evaporating monomers from the liquid jet. Tentatively, this observation can be attributed to the presence of a surface tension related activation barrier, specific to the evaporation of preexisting hydrophobic dimer inclusions.^2^

For photoelectron spectroscopy of liquid water microjets, the experimental challenge consists in operating the electron energy spectrometer in the presence of a plenitude of water vapor, threatening to change the metal surface vapor coverage and surface potentials unpredictably and leading to unstable electron energy analysis. For feasibility tests in the available molecular beams chamber, we used a refurbished hemispherical electron energy analyzer and the 21.2 eV photon line of a windowless helium discharge lamp. Differentially pumped light focusing capillaries helped to block the reverse flow of water vapor into the glow discharge region. Photon- or electron-exposed surfaces were made of noncharging copper–beryllium. The electron spectrometer detector was placed in a separate ultrahigh-vacuum chamber.

An initial issue of concern was that charging of the low-conductance water by photoelectron emission could alter the local electrostatic potential of the liquid jet. For monitoring the anticipated radiation charging, an electrically insulated water beam trap was set up for measuring the jet current. Unexpectedly, the microjets showed currents in the order of several nanoamperes, even when the UV lamp was turned off!^13^ After an extended literature search, it was understood that in ultrapure insulating water, with ionic dissociation of 10^–7^ M protons, the Θ-potential driven Debye layer of electrostatic surface charge separation has a thickness on the order of 1 μm. Incidentally, for the actual microjet velocities this is comparable to the thickness of the fluid dynamical “Prandtl” shear layer at the nozzle throat, with the consequence that the near surface H^+^/OH^–^ charge separation layer is optimally sheared off, leading to very strong charging in streaming pure water. This “electrokinetic” current flow is proportional to the jet speed. Minor pH value changes of ±0.1, resulting from minuscule, 1 ppb water contamination, can completely alter the electrokinetic charge and even affect charge polarity.^13^ The monitored electrokinetic current of low conductivity microjets was used to correct, via the Gauss Law of electrostatics, the instantaneous surface charge potential offset in the first photoelectron energy spectra of ultrapure water.^14^

**Figure 2:**
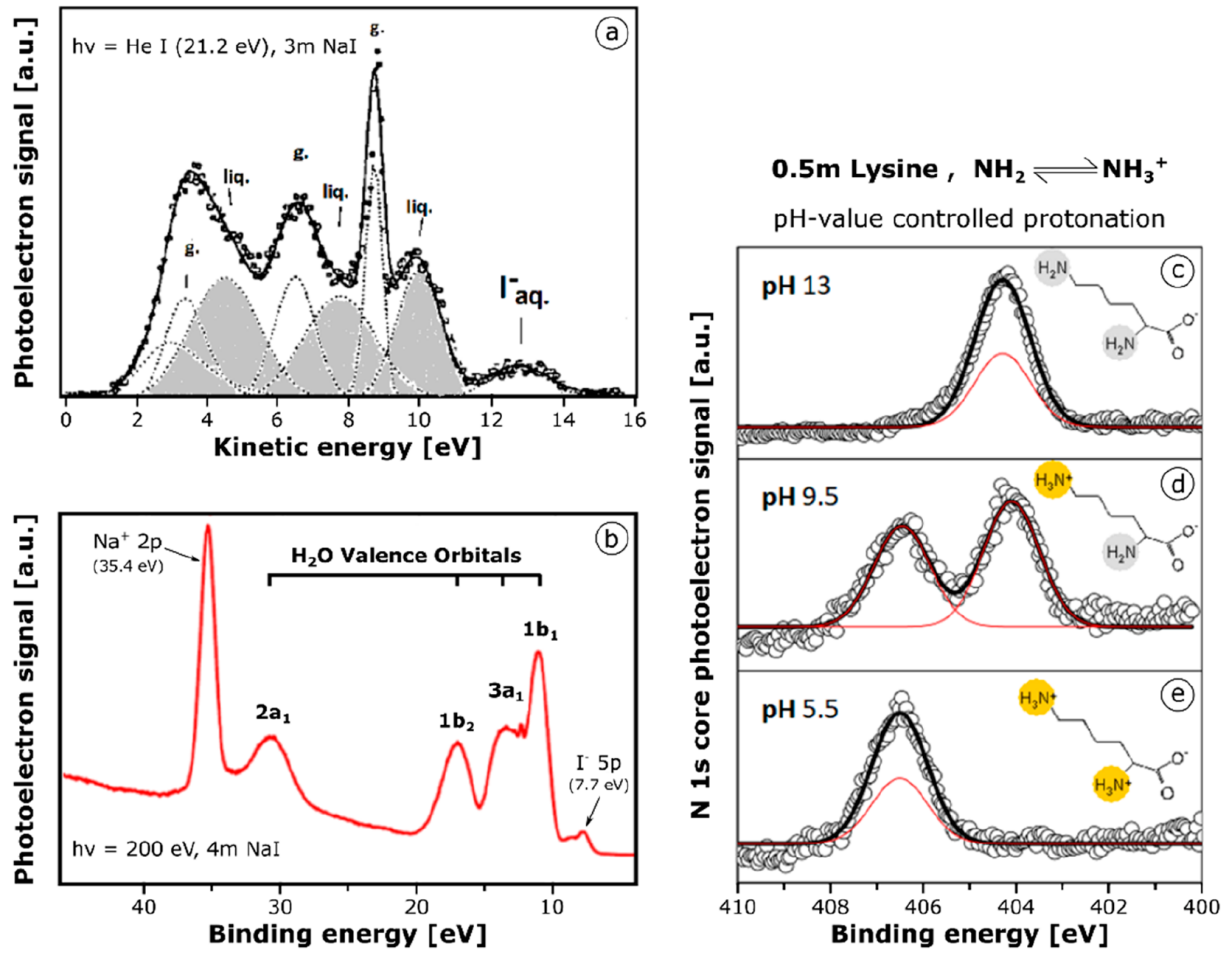
Valence electron and core electron photoelectron spectroscopy: (a) Early photoelectron spectrum of a 3 molal NaI solution, obtained with a He I lamps’ 21.2 eV radiation and using a 5 μm microjet. Overlapping gas-phase and liquid-phase contributions from the three outer valence water orbitals are obtained by numerical deconvolution shown by intermittent lines. The I^–^_aq_ peak doublet P_1/2_/P_3/2_ appears at 7.7 eV binding energy. (b) A 4 *m* NaI photoelectron spectrum obtained ten years later for 200 eV radiation in the optimized synchrotron radiation experiment, using a 20 μm microjet. Due to improved photon beam and electron energy spectrometer collimators and also for larger jet size and liquid temperature control to ∼5 °C, the gas phase contribution is almost negligible for this spectrum. All four water valence orbitals, including the “inner valence” 2a_1_ state at *E*_B_= 30.5 eV, are readily observed; the Na 2p^+^ peak of the solvated cation appears at 35.4 eV binding energy. A representative result for inner shell ionization (ESCA) photoelectron spectroscopy in chemical solutions is illustrated in panels c, d, and e. Inner shell X-ray photoelectron spectroscopy with soft X-ray, 480 eV photons allows sampling of the binding state of selected specific atoms in a chemical compound such as the N 1s nitrogen orbital in a 0.5 molal solution of the amino acid lysine. Because the pH value in the microjet liquid can be simply controlled, it is possible to watch the N 1s peak shift and peak amplitudes of the ongoing protonation of the two NH_2_ amino groups to NH_3_^+^, occurring for lysine at different values of p*K*_a2_ = 8.95 and p*K*_a3_ = 10.53. Parts of this figure were reproduced with permission from ref (19) (Copyright 2010 Elsevier) and ref (20) (Copyright 2007 American Chemical Society).

Alternatively, in slightly conducting liquid water, the electrokinetic charging of microjets is reduced to negligible levels by addition of small quantities of salt, ≥0.05 molal NaCl, for example. However, external electrical fields may induce charging of conducting jets, depending on the resistance–capacitance relation in the liquid jet and jet decay time. Unaware of this issue in the earliest microjet photoelectron measurements on salt-brines, we had followed Siegbahn’s (1973) suggestion^5^ for suppressing gas-phase photoelectron contributions by a superimposed electric field for leveling the (nonlocalized) gas phase peaks while leaving true solution-surface peaks intact. Not finding any halogen ion spectrum peak in difference measurements with pure water, we were misled to assume for some time that ions, perhaps, do not sit at the surface of water.^15^ Only after substantial improvements in the He I lamp collimation and in photoelectron sensitivity, we eventually observed the first genuine photoelectron peaks of solvated anions with He I, 22.1 eV photoexcitation at a microjet of 3 M NaI aqueous solution shown in Figure 2a.^16,17^

Even in this example, a large fraction of the gas phase water vapor signal is superimposed on the proper liquid-phase PES. These spectra can be deconvoluted into individual contributions from three gaseous H_2_O outer valence electron peaks (marked “g” in Figure 2a) and the corresponding liquid water valence electron peaks (light gray shaded and labeled “liq”). They appear strongly broadened in the liquid (unfortunately) and are shifted due to solvation by 1.4 eV with respect to the gas phase ionization energy levels. The aqueous iodide ion I^–^ doublet states P_1/2_/P_3/2_ (Δ*E* ∼ 1 eV) are observed as a broad twin peak at 7.7 eV average binding energy, which is ∼4 eV deeper than the known gas-phase electron-detachment energy of the I^**–**^ ion, confirming qualitatively the expectations of dielectric polarization models of ionic solvation.^5,17^

Hydrated cations, because of their larger vertical ionization energies, were not accessible with the limited helium I photon energy. However, third generation synchrotron radiation photon sources became available, with very high intensities and micrometer size beam focus ideally matching the water microjets. After some short, initial hesitation in view of the tremendous water vapor load, we were admitted to try out an optimized microjet PES apparatus for its performance on a Bessy II beamline. Following initial experimentation on a 100 eV beamline, a first set for all (stable) alkali halide cation–anion 1st vertical ionization energies was completed in 2005.^3,18^ Continuing optimization and access to a microfocus-beamline for up to 1 keV radiation led to further signal improvement and largely reduced gas contributions as illustrated, in Figure 2b, with a later measurement on a 4 molal NaI solution obtained at *h*ν = 200 eV.^19^ The spectrum extends over all valence peaks and identifies the binding energies of the Na^+^_aq_ 2p state, all four valence orbitals of liquid water, and I^–^_aq_.

We also readily observed the inner shell photoelectron spectra of carbon, nitrogen, or oxygen in the soft X-ray photon energy range, which can provide site-specific information on the chemical binding environment of a targeted atom.^5^ Thus, in water microjets, the pH value dependent protonation can be visualized microscopically as the instantaneous ionization state of a molecular subgroup. This is shown for 0.5 molal samples of the amino acid lysine, in Figure 2c–e, at three pH values of 13, 9.5, and 5.5.^20^ Lysine carries two NH_2_ groups, which are protonated to NH_3_^+^ groups at different values of p*K*_a2_ = 8.95 and p*K*_a3_ = 10.53. The N 1s core electron binding energy of 404.3 eV in the NH_2_ amino-groups changes upon protonation to 406.5 eV and is clearly visible as a chemical shift between two distinct N 1s core photoelectron peaks. The peak amplitude ratios represent the quantitative ratio between NH_3_^+^ and NH_2_, allowing straightforward microtitration. In analogous studies of more complex aqueous systems, even variations in composition from surface sites to bulk liquid could be analyzed by varying the photon energy and exploiting the increase of electron escape depth with the photoelectron energy.^21^

The flexible and sensitive PE spectroscopy with narrow bandwidth and tunable X-rays made possible a unique detection and quantification of unoccupied valence band orbitals by resonant Auger excitation photoelectron spectroscopy. Charge transfer to solvent (CTTS) excited states of closed shell anions (Cl^–^ or OH^–^), due to solvent polarization, appear in the aqueous solution near the ionization threshold, while the gaseous negative ions exist exclusively in a single orbital state. Visible in classical ultraviolet photometry, the strong CTTS absorption line is obscured by liquid water absorption bands but freely observable in inner shell resonant absorption. A completely unexpected result emerged from a search for CTTS excited states of OH^–^ with *h*ν = 532.8 eV resonant Auger PES, that revealed an unknown, ultrafast excitation energy transfer process of excited OH^–^_aq_ states to nearby liquid water.^4,22^ The near continuum CTTS can also function as a gateway for free solvated electron formation in water. This result was further explored in a new pump–probe liquid microjet experiment, with femtosecond laser excitation to transition states, and high harmonics generated 37.8 eV photons used for the first subsequent photoelectron measurement of the ionization energy for the short-lived solvated electron.^23^

Considering the diversity of chemical topics already studied in microjets in the past by the large number of players in the field, it seems difficult to forecast routes for future developments in water-in-vacuum studies. According to my own preferences, shaped microjet vacuum targets^24^ will play a role in answering questions involving angular distributions and of electron escape depth. New avenues will open up with time-resolved pump–probe molecular scattering and evaporation combined with ion-imaging photoionization detection techniques. Worth striving for, though perhaps impossible, would be increases in accuracy of vertical ionization energy measurements, to better than 1 meV, for direct microtests of thermodynamically derived electrochemical relations, such as Nernst equation dependences of electrical half-cell potentials and of the detailed structure of electrical double layers.
